# Contextual Assessments for Chronic Obstructive Pulmonary Disease Transition of Care Bundle Implementation Planning for the Reduce REVISITS Study: Rapid Sequential Explanatory Mixed Methods Approach

**DOI:** 10.2196/82078

**Published:** 2026-03-02

**Authors:** Mahima Akula, Kim Erwin, Leah Traeger, Hannah Pick, Fei Gao, Laura Damschroder, Valerie G Press

**Affiliations:** 1 Department of Medicine, University of Chicago, 5841 S Maryland Ave MC 2007, Chicago, IL, 60637, United States, +1 (773) 702-5170; 2 Institute of Design, Illinois Institute of Technology, Chicago, IL, United States; 3 Onda Collective, LLC, Chicago, IL, United States; 4 Implementation Pathways, Chelsea, MI, United States

**Keywords:** chronic obstructive pulmonary disease, COPD, readmission, revisits, transitions of care

## Abstract

**Background:**

Chronic obstructive pulmonary disease (COPD) affects more than 16 million US adults, many of whom experience high rates of acute care revisits (emergency department and hospital) after initial hospitalization. These frequent exacerbations, often due to failures in transitions of care (TOC), lead to lung function decline and premature mortality. While effective interventions exist to reduce readmissions, wide-scale implementation of COPD TOC programs remains limited. The National Institutes of Health–funded Reducing Respiratory Emergency Visits Using Implementation Science Interventions Tailored to Settings (REVISITS) study was designed to address this implementation gap by developing and implementing bundled COPD TOC programs across diverse US hospitals.

**Objective:**

This study aimed to conduct pre-implementation contextual assessments at US hospitals to guide the development of site-specific, evidence-based COPD TOC programs.

**Methods:**

We conducted pre-implementation contextual assessments using a novel semi-structured interview format that integrated the Consolidated Framework for Implementation Research (CFIR) with human-centered design approaches (ethnographic interviewing) to capture real-world experiences of COPD care across inpatient, outpatient, and home settings. We used a sequential explanatory mixed methods design in which pre-interview survey data completed by site leads informed and shaped the subsequent semi-structured interviews. Site leads, clinicians, organizational leaders, patients, and caregivers were interviewed. Interviews explored baseline COPD TOC practices, local resources, opportunities for improvement, as well as participant priorities from a menu of 12 evidence-based interventions (eg, pulmonary rehabilitation, patient navigation, and inhaler teaching). Rapid analysis methods identified intervention priorities across participant groups, along with perceived barriers and facilitators to implementation. Findings were shared with site leads to help guide their development of tailored COPD TOC programs.

**Results:**

Among 194 participants from 21 sites (42 site leads, 29 organizational leaders, 105 clinicians, and 18 patients or caregivers), the highest priority interventions identified during interviews were post–emergency department follow-up visits, education (inhaler technique, disease management, and action plan), and pulmonary rehabilitation. Reported barriers included clinician-level challenges (limited training, staffing, and time), patient-level challenges (social needs and physical burden of COPD), and system-level challenges (lack of standardization, limited resources, and cost). Key facilitators included the presence of dedicated staff and the availability of pre-existing programs or infrastructure. The 3 most commonly chosen interventions for implementation were patient education (eg, inhaler education and COPD action plans), medication reconciliation, and post-discharge care (eg, post-discharge visits and pulmonary rehabilitation).

**Conclusions:**

This study demonstrates how the integration of implementation science and human-centered design approaches can yield valuable insights, beyond what either field could obtain separately, during the pre-implementation phase of COPD TOC program implementation development. Contextual assessments that capture diverse views are instrumental in designing feasible and relevant interventions. Future work will explore how pre-implementation insights relate to post-implementation outcomes across participating sites.

## Introduction

Chronic obstructive pulmonary disease (COPD) affects more than 16 million US adults, many of whom experience high rates of acute care revisits (emergency department [ED] and hospital) after initial COPD hospitalizations [1-5]. These frequent exacerbations, often due to failures in transitions of care (TOC), lead to lung function decline and premature mortality [6]. In response, the Centers for Medicare and Medicaid Services launched the Hospital Readmission Reduction Program (HRRP), which includes COPD readmissions as a targeted outcome [7]. Additionally, the Centers for Medicare and Medicaid Services introduced the Bundled Payments for Care Improvement (BPCI) initiative, a voluntary program designed to encourage coordinated care and reduce costs associated with hospital readmissions [7-9]. These policies motivated many US hospitals to pursue interventions aimed at reducing COPD readmissions to avoid financial penalties. However, beyond a few published reports of successful TOC interventions, there is limited clarity regarding what constitutes a successful COPD TOC program [10-14]. To date, no systematic evaluation has been conducted on the types or consistency of care transition interventions used within or across hospitals since the implementation of the HRRP. As a result, wide-scale implementation of COPD TOC programs remains limited by fundamental gaps in effectiveness and implementation data [15-17].

The Reduce Respiratory Emergency Visits Using Implementation Science Interventions Tailored to Settings (REVISITS) study was designed to address these gaps by developing, implementing, and evaluating bundled COPD TOC programs across diverse US hospitals [18]. Before sites launched their tailored COPD TOC programs (comprised of 2‐3 evidence-based interventions), we conducted pre-implementation contextual assessments to identify baseline COPD TOC practices, local resources, and opportunities for improvement. These contextual assessments were intended to inform site-specific program development and promote successful implementation and sustainability.

## Methods

### Study Design and Setting

**Figure 1.**
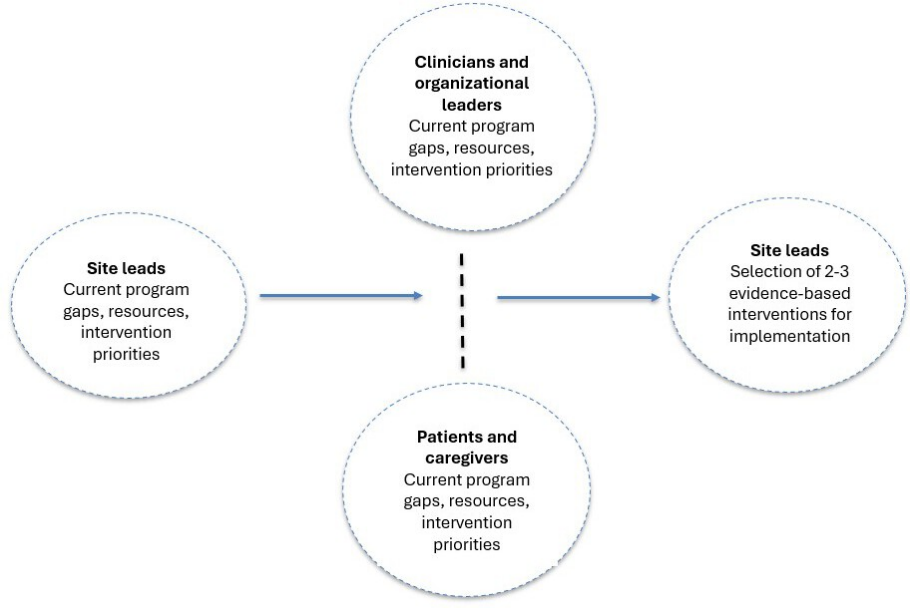
Pre-implementation contextual assessment approach used in the Reduce Respiratory Emergency Visits Using Implementation Science Interventions Tailored to Settings (REVISITS) study, a sequential explanatory mixed methods study conducted across 21 US hospitals to inform the design of chronic obstructive pulmonary disease transition of care programs. The figure outlines the stepwise discovery process integrating human-centered design and implementation science methods, including pre-interview surveys, multiparticipant interviews, rapid analysis, and site-specific implementation planning.

The Reduce REVISITS study used a sequential explanatory mixed methods design [19]. Site leads first completed a pre-interview survey to identify preliminary priorities, site characteristics, and contextual factors. These survey responses were then used to inform and tailor the subsequent semi-structured interviews, during which participants elaborated on and clarified their survey responses. This sequential approach allowed the qualitative interviews to build on the quantitative survey data, providing deeper insight into site-level priorities and implementation considerations. Using a structured, stepwise discovery process, we conducted pre-implementation contextual assessments at each participating site. These assessments were designed to help sites develop COPD TOC programs tailored to their specific needs and environment. Specifically, the assessments aimed to characterize each site’s local context, evaluate readiness for change, and identify desirable evidence-based COPD interventions for inclusion in the site’s TOC program (Figure 1).

Hospitals were recruited into this initial phase of the study from the Hospital Medicine Reengineering Network [20] and through the Society of Hospital Medicine [21]. The study aimed to enroll at least 20 hospitals to complete this phase to ensure sufficient power for the randomized trial in the following phase [18].

Each participating hospital designated 2 site leads responsible for leading local study-related COPD TOC intervention implementation efforts. The process began with site leads completing a brief survey (Multimedia Appendix 1), followed by in-depth interviews. During the interviews, site leads described the current state of COPD care at their organization, identified potential barriers and facilitators to implementation, and prioritized interventions for inclusion in their site’s TOC program. Site leads then identified additional key participants, including clinicians and organizational leaders, whose input would further inform the COPD TOC program implementation. Site leads were also encouraged to distribute flyers and engage in efforts to recruit patients hospitalized with a COPD exacerbation in the past year and their caregivers to the study.

### Data Collection Tools and Procedures

We developed data collection tools by human-centered design (HCD) ethnographic interviewing techniques, such as journey mapping, projective images, and concept ranking [22,23], with implementation science (IS) methodology, specifically constructs from the Consolidated Framework for Implementation Research (CFIR) [24-28].

Qualitative data collection was guided by a pragmatic research paradigm, which emphasizes generating actionable, contextually grounded insights to support real-world implementation planning. This paradigm aligned with the study’s objective to identify practical barriers, facilitators, and priorities that could directly inform the design of site-specific COPD TOC programs.

Site leads first completed a pre-interview survey on their site’s current practices and resources that informed the patient or caregiver, clinician, and organizational leader interviews. All participants were interviewed using semi-structured guides. During interviews, participants interacted with a structured slide deck to prompt reflection and discussion. For example, patients were presented with 11 projective images and asked to select 2 that represented their experiences with COPD, while clinicians ranked 12 evidence-based interventions by priority. Interviews were conducted by members of the study team trained in qualitative interviewing, including individuals with backgrounds in medicine (VGP), HCD (KE), health services research (MA and LT), behavioral science (HP and FG), and IS (LD). We acknowledge that these professional backgrounds may influence data collection and interpretation. To mitigate bias, interviewers used consistent probes and documented reflections during and after interviews. Each interview was attended by at least 2 members of the study team (MA, LT, KE, HP, and FG). One team member conducted the interview while the second took detailed notes, facilitating cross-validation and supporting subsequent analysis. No interviewers had previous relationships with any participants. Interviewers introduced themselves by name and study role at the start of each interview and emphasized that participation was voluntary and confidential.

Site leads at each participating hospital assisted with identifying eligible potential participants and provided the study team with contact information for these individuals. Site leads also distributed study flyers to recruit patients hospitalized for a COPD exacerbation in the previous year and their caregivers. The study team used a standardized recruitment protocol across sites, which included initial email or phone outreach, confirmation of interest, and scheduling of interviews. To ensure consistency, all recruitment communications followed a uniform script.

All interviews were conducted virtually via Zoom (Zoom Communications) or a recorded telephone call. For patients and caregivers with limited technology access, study materials were mailed, and phone interviews were offered as an alternative to Zoom. No in-person interviews were conducted.

### Data Types and Quantitative or Qualitative Approaches

Using a sequential explanatory mixed methods approach, we collected both qualitative and quantitative data. Quantitative data included structured ratings and rankings (eg, intervention priorities and readiness scores) and were summarized using descriptive statistics (means and medians) to assess inter- and intrasite variation in priorities and context. Qualitative data were analyzed using a qualitative descriptive design to summarize participants’ experiences and perspectives. This approach aligns with the pragmatic paradigm and the study’s objective to rapidly generate actionable insights rather than interpret lived experience from a philosophical standpoint. We adopted a rapid qualitative analysis approach, which allowed data to be synthesized and reported to site leads in time to inform the development of COPD TOC programs.

Consistent with a sequential explanatory mixed methods design, quantitative survey data were first used to shape and tailor the subsequent qualitative interviews. Integration occurred at multiple points: (1) during data collection, where pre-interview survey responses informed interview probes and areas for follow-up; (2) during analysis, where quantitative intervention rankings were compared with qualitative descriptions of barriers and facilitators; and (3) during interpretation, where findings across data sources were synthesized to develop site-specific contextual needs. This integrated understanding directly informed the site-specific implementation planning discussions.

### Rapid Analysis and Summary Development

Interview data from site leads, organizational leaders, clinicians, patients, and caregivers were collected and analyzed by the study team (MA) using a rapid analysis approach. This analysis resulted in the formation of a “priority summary table,” which ranked interventions based on the number of participants who identified them as high priorities, and a “resource table,” which listed available resources that could support implementation of priority interventions. These materials were compiled to inform intervention planning for each site.

### Implementation Planning Discussions and Reports

Post analysis, site-specific findings, and intervention-specific evidence summaries were presented to site leads during facilitated implementation planning discussions led by the principal investigator (VGP). Based on this information, site leads were asked to select 2‐3 interventions to implement as part of their site’s COPD TOC program. Following these discussions, the study team (MA and LT) created tailored “site implementation reports” that site leads could use when planning implementation of their selected interventions. These reports included key takeaways from interview data, such as barriers and facilitators to implementation, priority and resource summary tables, and notes from the implementation planning discussion. Each report also included a “workbook” component to guide sites in planning implementation steps and collecting baseline quantitative data (Multimedia Appendix 2).

### Ethical Considerations

This study was reviewed and approved by the University of Chicago Institutional Review Board (IRB; IRB20-0373). All participants provided oral informed consent before participation in semi-structured interviews conducted via Zoom or recorded phone call. Site leads at each participating hospital assisted in identifying eligible stakeholders and shared contact information with the study team, who then contacted potential participants directly.

All research activities were conducted by trained study personnel following a standardized, written protocol. Participant confidentiality was rigorously maintained: all site-level data were fully deidentified, hospital data were stored in REDCap (Research Electronic Data Capture; Vanderbilt University), and interview data were housed on a secure, HIPAA (Health Insurance Portability and Accountability Act)–compliant shared drive managed by the University of Chicago Center for Research Informatics. Study ID numbers were used in place of personal identifiers. Identifiable contact information collected for recruitment and compensation was stored separately from study data and will be destroyed, along with audio files, after study completion.

Participants received compensation approved by the IRB. Patients and caregivers received a US $25 gift card, and clinicians and organizational leaders received a US $10 gift card. Site leads who supported implementation at participating hospitals received an honorarium of US $3500 per year of participation. Raw data will not be shared outside the study investigators, research personnel, and approved external collaborators covered under the University of Chicago IRB.

## Results

### Site Characteristics

In total, 21 hospital sites were enrolled in the first phase of the Reduce REVISITS study. Approximately two-thirds (13/21) were academic hospitals, and one-third (8/21) were community hospitals. There was considerable geographic diversity among enrolled sites, with the Midwestern (7/21), Northeastern (6/21), and Southern (6/21) regions of the United States roughly equally represented. However, only 2 sites were enrolled from the Western region (Table 1).

**Table 1.** Characteristics of the 21 US hospital sites enrolled in the pre-implementation phase of the Reduce REVISITS^a^ study, including hospital type and region.

Characteristic	Sites (n=21), n (%)
Academic	13 (61.9)
Community	8 (38.1)
Northeast	6 (28.5)
Midwest	7 (33.3)
South	6 (28.5)
West	2 (9.5)

a REVISITS: Respiratory Emergency Visits Using Implementation Science Interventions Tailored to Settings.

Across the 21 sites, a total of 194 participants were interviewed. Among these participants, 176 were hospital-affiliated individuals (site leads [n=42], organizational leaders [n=29], and clinicians [n=105]), and 18 were patients or caregivers (n=16 and 2, respectively).

### Participant Characteristics

Overall, participant demographics varied by participant group (Table 2, Supplementary Table S1 in Multimedia Appendix 3). Hospital-affiliated individuals tended to be younger than patients and caregivers. When obtaining demographic information, we asked participants what gender they currently identify with, and gender distribution varied by participant group. Although side leads were roughly split between males and females (22 females and 20 males), most organizational leaders (21/29) were male, while most clinicians (70/105) and patients and caregivers (13/18) were female. Racial diversity was limited and also varied by participant group. Most notably, there were significant socioeconomic and educational disparities. Most hospital-affiliated individuals (149/176) reported household incomes higher than US $100,000, whereas approximately two-thirds (11/18) of patients and caregivers reported an average household income of less than US $40,000. The majority of site leads, organizational leaders, and clinicians (140/176) held a master’s degree or higher, whereas most patients and caregivers (14/18) had not attained a bachelor’s degree.

**Table 2.** Demographic characteristics of the 194 stakeholders interviewed during pre-implementation contextual assessments for the Reduce REVISITS^a^ study, including site leads, clinicians, organizational leaders, and patients or caregivers across 21 US hospitals.

Characteristics	Patients+ caregivers (n=18)	Site leads (n=42)	Organizational leaders (n=29)	Clinicians (n=105)
Age (y)				
Mean (SD)	65.83 (10.62)	42.10 (8.45)	47.61 (9.52)	43.36 (9.25)
Median (IQR)	67.00 (9.5)	40.50 (12.75)	46.00 (9.5)	41.50 (13)
Range (minimum-maximum)	47 (40‐87)	32 (29‐61)	36 (33‐69)	42 (26‐68)
Declined to answer, n (%)	0 (0)	0 (0)	1 (3.45)	7 (6.67)
Gender identity, n (%)				
Female	13 (68.42)	22 (52.38)	8 (27.59)	70 (66.67)
Male	5 (26.32)	20 (47.62)	21 (72.41)	35 (33.33)
Race, n (%)				
American Indian or Alaskan Native	0 (0)	0 (0)	0 (0)	0 (0)
Asian	0 (0)	11 (26.19)	5 (17.24)	14 (13.33)
Black or African American	5 (26.32)	1 (2.38)	0 (0)	5 (4.76)
Native Hawaiian or Other Pacific Islander	0 (0)	0 (0)	0 (0)	1 (0.95)
White	11 (57.90)	26 (61.90)	23 (79.31)	73 (69.52)
Other	1 (5.26)	3 (7.14)	1 (3.45)	7 (6.67)
Declined to answer	1 (5.26)	1 (2.38)	0 (0)	5 (4.76)
Ethnicity, n (%)				
Hispanic or Latino	0 (0)	1 (2.38)	1 (3.45)	4 (3.81)
Not Hispanic or Latino	18 (100)	39 (92.86)	28 (96.55)	97 (92.38)
Declined to answer	0 (0)	2 (4.76)	0 (0)	4 (3.81)
Average household income (US $), n (%)				
Less than 20,000	9 (47.37)	0 (0)	0 (0)	0 (0)
20,000-40,000	2 (10.53)	0 (0)	0 (0)	0 (0)
40,000-60,000	1 (5.26)	1 (2.38)	0 (0)	1 (0.95)
60,000-80,000	0 (0)	1 (2.38)	0 (0)	4 (3.81)
80,000-100,000	0 (0)	1 (2.38)	2 (6.9)	2 (1.9)
More than 100,000	2 (10.53)	35 (83.33)	26 (89.66)	88 (83.81)
Declined to answer	4 (21.05)	4 (9.52)	1 (3.45)	10 (9.52)
Highest level of education, n (%)				
No formal educational credential	3 (15.79)	0 (0)	0 (0)	0 (0)
High school diploma or equivalent	6 (31.58)	0 (0)	0 (0)	0 (0)
Some college, no degree	4 (21.05)	1 (2.38)	0 (0)	0 (0)
Associate’s degree	1 (5.26)	1 (2.38)	0 (0)	10 (9.52)
Bachelor’s degree	3 (15.79)	3 (7.14)	1 (3.45)	20 (19.05)
Master’s degree	0 (0)	6 (14.29)	3 (10.34)	18 (17.14)
Doctor or professional degree	0 (0)	31 (73.81)	25 (86.21)	57 (54.29)
Other	1 (5.26)	0 (0)	0 (0)	0 (0)

a REVISITS: Respiratory Emergency Visits Using Implementation Science Interventions Tailored to Settings.

### Interventions Identified as Priorities by Interview Participants

Across all 194 participants, the most frequently prioritized interventions were post–hospital or ED follow-up visits (90/194, 46%), inhaler education (78/194, 40%), and COPD education (66/194, 34%). Pulmonary rehabilitation was similarly prioritized (66/194, 34%), followed by COPD action plan (61/194, 31%), and patient navigation (60/194, 31%). The least frequently prioritized interventions included remote monitoring and reporting, advanced care planning, and spirometry. High-priority interventions were similar across hospital-affiliated and patient participants, although hospital-affiliated participants more strongly prioritized post–hospital or ED follow-up visits (84/176 hospital-affiliated vs 6/18 patient participants), inhaler education (73/176 hospital-affiliated vs 5/18 patient participants), and COPD action plan (58/176 hospital-affiliated vs 3/18 patient participants).

There was general agreement across participant groups about both the most and least prioritized interventions (Table 3). For example, post–hospital or ED follow-up visit, COPD education, and COPD action plan were frequently selected as top priorities, while remote monitoring and reporting, advanced care planning, and spirometry were less favored. However, there was more participant variation for interventions that were only moderately prioritized. For example, pulmonary rehabilitation was prioritized more often by site leads and patients or caregivers than by organizational leaders and clinicians. The COPD action plan was the top-ranked intervention among clinicians, although it was not equally prioritized by other groups (Table 5).

**Table 3.** Evidence-based chronic obstructive pulmonary disease transitions of care interventions selected by site leads at 21 US hospitals during the pre-implementation phase of the Reduce REVISITS^a^ study, shown alongside corresponding participant-reported priority counts.

	Frequency, n (%)
Selected interventions (number of sites)	
Inhaler education	20 (95.24)
Post-ED^b^ follow-up visit	13 (61.9)
COPD^c^ action plan	12 (57.14)
Medication reconciliation	9 (42.86)
Pulmonary rehabilitation	6 (28.57)
Patient navigation	6 (28.57)
Other COPD education	4 (19.05)
Smoking cessation education	4 (19.05)
Advanced care planning	0 (0)
Spirometry	0 (0)
Remote monitoring and reporting	0 (0)
Priority intervention (number of participants)	
Post-ED follow-up visit	90 (46.39)
Inhaler education	78 (40.21)
COPD education	66 (34.02)
Pulmonary rehabilitation	66 (34.02)
COPD action plan	61 (31.44)
Patient navigation	60 (30.93)
Medication reconciliation	57 (29.38)
Smoking cessation education or treatment	53 (27.32)
Remote monitoring and reporting	27 (13.92)
Advanced care planning	15 (7.73)
Spirometry	14 (7.22)

a REVISITS: Respiratory Emergency Visits Using Implementation Science Interventions Tailored to Settings.

b ED: emergency department.

c COPD: chronic obstructive pulmonary disease.

### Participant-Identified Barriers and Facilitators

During interviews, site leads, organizational leaders, and clinicians identified a range of barriers and facilitators related to COPD TOC interventions (Table 4). Barriers occurred at the clinician, patient, and systems levels. Clinician-level barriers included a lack of staff, bandwidth, time, and lack of clinician education. Patient-level barriers included social determinants (eg, transportation, socioeconomic status, and technology literacy) as well as physical limitations (eg, poor vision and mobility issues). System-level barriers included a lack of standardization, inadequate resources (eg, placebo inhalers), shifts in care delivery (eg, nebulizer use), and cost-related issues (eg, pulmonary rehabilitation reimbursement and hiring patient navigators).

**Table 4.** Qualitative themes describing clinician-level, patient-level, and system-level barriers and facilitators to implementing chronic obstructive pulmonary disease transitions of care interventions, based on multiparticipant interviews conducted at 21 US hospitals during the pre-implementation phase of the Reduce REVISITS^a^ study.

TOC^b^ interventions	Representative CFIR^c^ domains and subdomains with quotes	
	Barriers	Facilitators
Inhaler education	Inner Setting: Work Infrastructure “We unfortunately don’t have those dummy inhalers [to] educate [patients].” *-*RT^d^ Individuals: Innovation Deliverers “I have no idea how patients get inhaler education. It’s not from me. I don’t really know how to give that education.” -SL^e^	Inner Setting: Work Infrastructure “We have a respiratory therapist whose job is education, and she is an asset.” -SL Individuals: Innovation Deliverer “Previous to [the pulmonary navigator], I was just going, ‘Puff, puff,’ to both of them. And he taught me how to use it.” -Patient
COPD^f^ education	Inner Setting: Work Infrastructure “It’s really hard to have the time to sit with the patient and discuss [education] in the hospital.” -Physician Individuals: Innovation Recipients “My eyes aren’t real good right now. So my glasses still ain’t letting me read real good.” -Patient	Inner Setting: Work Infrastructure “[The nurse-led education service] get this consult, and then they have a team of nurses that basically meet with the patient and family one-on-one.” -RN^g^ Individuals: Innovation Deliverer “The most valuable part [of treatment was] [the pulmonary navigators]’s directions on how to deal with all of it.” -Patient
COPD action plan	Innovation: Innovation Design “Some patients [are] discharged without a clear COPD action plan. Often it should be tucked in as part of their [after visit summary], but they leave with a 30-page document.” -Physician Inner Setting: Work Infrastructure “Just having enough staff to take care of patients is a problem. So whether the [COPD action plan] would be able to happen, I think it probably wouldn’t.” *-*Care Coordinator	Innovation: Innovation Complexity “I feel like we see [the COPD action plan as] the low-hanging fruit one that’s relatively easy to do.” -Physician Innovation: Relative Advantage “I have [a COPD action plan] on my refrigerator. [When] you’re in the beginning of the yellow, then you might think, ‘Oh, okay. It’s okay. It’ll get better.’ But really, that’s the starting of it go into the red.” -Patient
Smoking cessation and/or treatment	Inner Setting: Work Infrastructure “Whether they hand them a brochure or sit with the patient and have a 10-minute conversation, it’s very dependent on the therapist.” -RT Individuals: Innovation Recipient “I used some of the patches, but they didn’t really work because when I came home [from the hospital], my insurance wouldn’t pay for them.” -Patient	Inner Setting: Available Resources “There’s a smoking cessation clinic. Patients can get nicotine patches or resources to stop smoking.” -Physician Inner Setting: Work Infrastructure Deliverers “I’m trying to free up some full-time equivalents so that I can create some tobacco treatment specialists to go to the bedside.” -OL^h^
Medication reconciliation	Inner Setting: Work Infrastructure “[Medication reconciliation]’s owned by nursing and physicians here just because we simply don’t have the pharmacy bandwidth to own that process.” -Pharmacist Outer Setting: Policies and Laws “It can be an hours-long process of trying to get approval for [the inhaler] or finding the right [one] that’s on formulary.” -SL	Inner Setting: Information Technology “Our outpatient pharmacy just went online with Epic so that our nursing staff and physicians can see where in the process meds to beds are.” -Pharmacist Individuals: Innovation Deliverers “The transition-of-care pharmacist is extremely helpful. The pharmacists have a keen eye for what somebody’s insurance will cover and what they won’t.” -OL
Advanced care planning	Inner Setting; Work Infrastructure “Our palliative service is really involved, but they are already over-maxed.” -Physician Individuals: Capability ”There comes a point where it would be nice if there were professional people that were telling him this rather than his son telling him that the trajectory is not necessarily positive. I’m reluctant to bring it up with him because it increases his anxiety.” -Caregiver	Inner Setting: Information Technology Infrastructure “And then for the high risk [patients], we get that alert for [a] care conference screening, [which] sometimes [includes] discussing palliative care.” -Care Coordinator Inner Setting: Work Infrastructure “We do have a pretty robust advanced care planning initiative. [We have] people who are specifically hired to do advanced care planning for a good chunk of our inpatients.” -SL
Post-ED^i^ follow-up	Inner Setting: Work Infrastructure “[Trying to get patients an appointment is] a very labor-intensive process. One of our biggest frustrations, I would say, as hospitalists trying to discharge patients is that we have no help in making these follow-up appointments.” -SL Outer Setting: Local Conditions “I got a insurance but they don’t pay for transportation and stuff like that, I have to catch the bus, get on the train, and run from the train station to [the hospital], and I have to stop and rest because I get short of breath.” -Patient	Inner Setting: Available Resources “We have a transition of care clinic, which is pretty great. If you put in the order, they’re good about going bedside to the patient, handing them information related to their discharge diagnosis and then the actual appointment in hand.” -Physician Inner Setting: Work Infrastructure “There’s been a very small improvement in [clinician availability] because we’ve hired two pulmonologists that actually do clinics now.” -SL
Pulmonary rehabilitation	Outer Setting: Financing “There are only two to three pulmonary rehab facilities in [the region]. And it’s very difficult for patients to get to, and it is because the Centers for Medicare and Medicaid Services reimbursement for pulmonary rehab is abysmally low.” -Physician Individuals: Innovation Recipients “I think [pulmonary rehab] was very good for me [in the past]. I think would be good for me now too [but] I don’t want to ask my son because it’s like you’re there for two hours and then to travel is another hour.” -Patient	Outer Setting: Partnerships and Connections “To try address [transportation barriers], we are looking at tele pulmonary rehab programs that are currently being offered by external vendors.” -Physician Inner Setting: Information Technology “Pulmonary rehab [is] going to be included in the new COPD discharge order set that’s going to roll out later this month.” -SL
Patient navigation	Innovation: Innovation Cost “But unfortunately, we only have one [nurse navigator]. We’re hoping that [the system] [will] hire more nurse navigators, but they have not because, unfortunately, they have not proven their return on investment.” -OL Outer Setting: Policies and Laws ”Yeah. I wish I had one of these little portable oxygen machine that I could tote on my shoulders. It’s all up to my insurance company.” -Patient	Individuals: Innovation Deliverer “So in my role [as a navigator], I do catch a lot when I go to rounds. We do COPD education. If there are discharge needs, then I take care of that. Then [I do] a follow-up phone call at least 48 hours after they’re discharged.” -RT Individuals: Innovation Deliverer “[The pulmonary navigator] is the most valuable person I’ve met [because of] his knowledge and his ability to translate medical terms into laypeople’s terms so that I can understand it and was not frightened by any of the approach or the medication.” -Patient
Spirometry	Inner Setting: Work Infrastructure “With COVID though, with the lab being shut down for quite a while, the backlog of people who needed pulmonary function testings got so large that we’re booking three months out.” -Physician Individuals: Innovation Recipients “But in my opinion, patients are not really aggressive about using spirometry because it’s a little aggravating and it’s easily forgotten.” -Care Coordinator	Inner Setting: Work Infrastructure “We kind of piloted [an outreach program] on a very small scale. Once we identified our COPD population, [we] just pulled some [patients who] did not have spirometry and reached out [to schedule pulmonary function testings]. We learned that you can cold-call patients, and that patients do respond and come in.” -Physician Inner Setting: IT Infrastructure “[In the portal they ask] patients to fill out a questionnaire, and then based on the results of the questionnaire, it lets them self-schedule to get their spirometry done.” -Physician
Remote monitoring and reporting	Inner Setting: Work Infrastructure “We do have this remote monitoring system but as I mentioned, it’s just not utilized. And then there’s only so many kits that we have.” -SL Individuals: Innovation Recipients “I think a lot of the elderly patients find the tablet and entering all this to be a little overwhelming. They don’t want to deal with it.” -Care Coordinator	Inner Setting: Work Infrastructure “I do know that we started an at-home monitoring system during COVID. Originally, it was designed just to be kind of post-COVID hospitalizations, but then the hospital expanded it to heart failure and COPD.” -Physician Individuals: Innovation Recipients “But I bought myself an oximeter because you can’t see the numbers of your oxygen unless you’ve got a machine to show you the numbers. I’m the guy that wants to know when I can’t breathe, what’s my number now?” -Patient

a REVISITS: Respiratory Emergency Visits Using Implementation Science Interventions Tailored to Settings.

b TOC: transitions of care.

c CFIR: Consolidated Framework for Implementation Research.

d RT: respiratory therapist.

e SL: site lead.

f COPD: chronic obstructive pulmonary disease.

g RN: registered nurse.

h OL: organizational leader.

i ED: emergency department.

The most prominent facilitators included having a dedicated staff or dedicated team (eg, COPD educator, pulmonary navigator, nurse education team, and TOC pharmacist), existing infrastructure (eg, transition of care clinic, smoking cessation clinic, and electronic health record), and ongoing quality improvement (QI) initiatives and protocols (eg, advanced care planning initiative, virtual pulmonary rehabilitation, and telehealth program).

Patient perspectives often aligned with those of clinicians, especially regarding challenges with follow-up visits and pulmonary rehabilitation. Both groups cited lack of clinician availability and transportation as key barriers. At one site, both groups emphasized the benefit of a dedicated pulmonary navigator. However, there was some disagreement around the utility of printed materials. Some patients and clinicians questioned their usefulness (eg, due to poor eyesight or redundancy), while others endorsed them, particularly the COPD action plan, which some patients kept visible at home and some clinicians described as efficient. These differing perspectives were discussed during intervention selection with site leads.

### Interventions Selected for Implementation by Site Leads

Inhaler education was the most frequently selected intervention for implementation, chosen by nearly all sites (20/21). Other commonly selected interventions included posthospital or ED follow-up visits (13/21) and COPD action plan (12/21). Fewer sites chose to implement medication reconciliation (9/21), pulmonary rehabilitation (6/21), patient navigation (6/21), COPD education (4/21), and advanced care planning (4/21). No sites chose advanced care planning, spirometry, and remote monitoring and reporting. While some participants listed spirometry and remote monitoring and reporting as priorities, site leads were discouraged from selecting these interventions as they could not be randomized to virtual or in-person delivery. Site leads were encouraged by the study team to include these as supplementary interventions if they also selected 2 eligible interventions compatible with the study design.

Overall, intervention selections aligned with the priorities expressed by site participants (Tables3  5). In total, 5 of the 6 interventions ranked as priorities were chosen for implementation. An exception is COPD education, which was ranked high as a priority but was less frequently selected for implementation.

**Table 5.** Interventions ranked as high priority by 194 multistakeholder interview participants (site leads, clinicians, organizational leaders, and patients or caregivers) across 21 US hospitals in the pre-implementation phase of the Reduce REVISITS^a^ study. Values represent the number and percentage of participants within each participant type who identified the intervention as a high priority.

Intervention	All (n=194), n (%)	Site leads (n=42), n (%)	Organizational leaders (n=29), n (%)	Clinician (n=105), n (%)	Patients+caregivers (n=18), n (%)
Post-ED^b^ follow-up visit	90 (46.39)	33 (78.57)	12 (41.38)	39 (37.14)	6 (33.33)
Inhaler education	78 (40.21)	27 (64.29)	7 (24.14)	39 (37.14)	5 (27.78)
COPD^c^ education	66 (34.02)	9 (21.43)	10 (34.48)	40 (38.1)	7 (38.88)
Pulmonary rehabilitation	66 (34.02)	24 (57.14)	4 (13.79)	31 (29.52)	7 (38.88)
COPD action plan	61 (31.44)	9 (21.43)	4 (13.79)	45 (42.86)	3 (16.67)
Patient navigation	60 (30.93)	26 (61.9)	8 (27.59)	20 (19.05)	6 (33.33)
Medication reconciliation	57 (29.38)	19 (45.24)	5 (17.24)	32 (30.48)	1 (5.56)
Smoking cessation education or treatment	53 (27.32)	20 (47.62)	4 (13.79)	28 (26.67)	1 (5.56)
Remote monitoring and reporting	27 (13.92)	7 (16.67)	2 (6.9)	17 (16.19)	1 (5.56)
Advanced care planning	15 (7.73)	2 (4.76)	2 (6.9)	10 (9.52)	1 (5.56)
Spirometry	14 (7.22)	0 (0)	0 (0)	14 (13.33)	0 (0)

a REVISITS: Respiratory Emergency Visits Using Implementation Science Interventions Tailored to Settings.

b ED: emergency department.

c COPD: chronic obstructive pulmonary disease.

## Discussion

### Principal Findings

This study integrated HCD and IS methods to conduct multiparticipant pre-implementation contextual assessments at 21 hospitals. The most frequently prioritized interventions were mostly related to post-discharge care (eg, post-discharge follow-up visits, pulmonary rehabilitation, and post-discharge patient navigation) and patient education (eg, inhaler education, general COPD education, and COPD action plan). Barriers to implementation occurred at the patient level (social and physical challenges), clinician level (resources: time, staffing, and training), and system (lack of standardization and/or resources to support COPD TOC programs). Key facilitators included harnessing available resources and activities, such as having dedicated staff, using existing infrastructure, and partnering with ongoing QI initiatives. Importantly, intervention selections by site leads generally aligned with the priorities identified by other participants at the same site, suggesting strong participant engagement and feasibility.

These findings align with previous studies identifying education, medication management, and structured follow-up as central components of effective COPD TOC programs. This study extends the existing literature by demonstrating the value of integrating HCD with IS methods as a structured, replicable approach for translating contextual data into actionable implementation decisions during the pre-implementation phase. The integration of HCD and IS enabled the identification of both universal priorities and site-specific constraints that directly informed intervention selection and implementation planning. The inclusion of patients and caregivers allowed the identification of accessibility barriers that are often overlooked when relying solely on clinician or administrative perspectives. Additionally, the use of rapid analytic techniques enabled timely translation of qualitative data into site-specific reports, demonstrating a pragmatic approach for supporting real-world implementation planning in multisite QI initiatives.

Variation in intervention prioritization across participant groups highlights the importance of including multiple perspectives in intervention planning. Site leads’ reliance on existing staff and infrastructure as facilitators also aligns with IS frameworks emphasizing adaptability, compatibility, and resource fit [24-28]. These findings underscore the value of blending HCD and IS in identifying contextual barriers and facilitators.

This study has several limitations. While there was considerable geographic diversity among sites, there was limited representation of hospitals in the Western region of the United States compared with the other regions, and community hospitals were slightly less represented compared with academic hospitals; notably, no Veterans Affairs hospitals enrolled. Patient and caregiver recruitment was limited due to reliance on site leads for recruitment and low technology access among patients. Additionally, the use of snowball sampling may have overrepresented clinicians already engaged in QI, and nearly half of the clinician participants were physicians, potentially limiting perspectives from other health professionals. Due to time constraints, such that data analysis was needed to move to the implementation phase, we used a rapid analytic approach to summarize site-specific interview data. We implemented several quality assurance strategies, including standardized note-taking templates, a uniform analytic process, and principal investigator (PI) review of all transcripts, to preserve analytic rigor. However, no formal coding of transcripts was conducted, and the majority of the analysis was performed by a single analyst, which may introduce interpretive bias.

Another important potential source of bias is regarding the COPD TOC interventions. While the study team used rigorous methods to identify evidence-based COPT TOC interventions, including reviewing the literature (PubMed and Google Scholar), reviewing scoping and systematic reviews [29-31], and team-based expertise (eg, PI was lead or senior author on evidence reports) [16,17], it is possible that interventions were missed. However, participants could prioritize any intervention when completing the interviews. In addition, bias related to the intervention chosen may have been introduced during the final site lead interview or discussion. The information regarding summaries of the evidence supporting COPD TOC interventions was presented by the study PI during these implementation planning discussions, and hence, this summary and any additional input from the PI may have influenced intervention selection. For example, inhaler education has been an area of previous research led by the PI, which may have influenced site lead decision-making. However, concern for this limitation is mitigated by the additional data that inhaler education was consistently identified as a top priority across participant groups during the interviews before this session with the PI, and the PI was not present for any of the participant interviews. This suggests that prioritization of inhaler education and other interventions largely reflected the participants’ perspectives rather than investigator influence. Additionally, although COPD education was frequently identified as a priority by participants, it was selected less often by site leads. This may reflect the study team’s guidance to prioritize specific, measurable education-related interventions or the tendency for site leads to bundle multiple education components together. However, most interventions were chosen by site leads by integrating prespecified priorities in conjunction with site-level feasibility.

### Conclusion

Integrating HCD and IS methods during the pre-implementation phase can enhance the relevance and feasibility and possibly the sustainability of hospital-based COPD TOC interventions. Perspectives from patients, clinicians, and organizational leaders help identify both universal priorities and site-specific constraints, enabling tailored program design. These findings illustrate a replicable and scalable model for designing contextually grounded TOC programs in COPD and other chronic conditions. Future work will assess whether these pre-implementation insights predict implementation fidelity, reach, and sustainability as well as patient outcomes across sites in subsequent phases of the Reduce REVISITS study.

## Supplementary material

10.2196/82078 Multimedia Appendix 1 Site lead survey.

10.2196/82078 Multimedia Appendix 2 Example site implementation plan workbook.

10.2196/82078 Multimedia Appendix 3 Participant demographics of hospitalized adults with chronic obstructive pulmonary disease across sites participating in the Reduce REVISITS (Respiratory Emergency Visits Using Implementation Science Interventions Tailored to Settings) study. Values represent aggregated demographic variables provided by sites with available data during the baseline period (n=10,697) and the implementation period (n=2,745). Demographic indicators include age, gender identity, race, ethnicity, insurance coverage, and smoking status. Only sites that submitted demographic data are included.

## ABBREVIATIONS

BPCI: Bundled Payments for Care Improvement
CFIR: Consolidated Framework for Implementation Research
COPD: chronic obstructive pulmonary disease
ED: emergency department
HCD: human-centered design
HIPAA: Health Insurance Portability and Accountability Act
HRRP: Hospital Readmission Reduction Program
IRB: institutional review board
IS: implementation science
PI: principal investigator
QI: quality improvement
REDCap: Research Electronic Data Capture
REVISITS: Respiratory Emergency Visits Using Implementation Science Interventions Tailored to Settings
TOC: transitions of care
