# Supplementary figures and images for: Contextual Assessments for Chronic Obstructive Pulmonary Disease Transition of Care Bundle Implementation Planning for the Reduce REVISITS Study: Rapid Sequential Explanatory Mixed Methods Approach
Source: JMIR Hum Factors. 2026 Mar 2;13:e82078. doi: 10.2196/82078 (PMC12954717; doi:10.2196/82078)

**Appendix A: Site Lead Survey
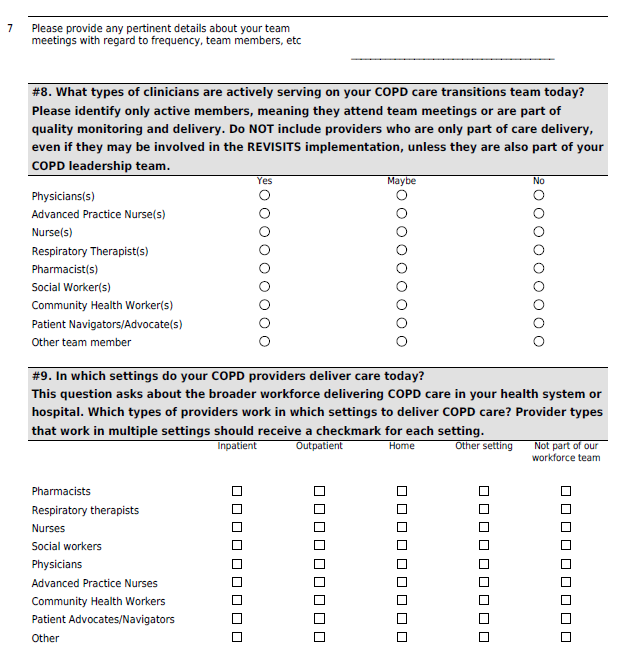
**

**
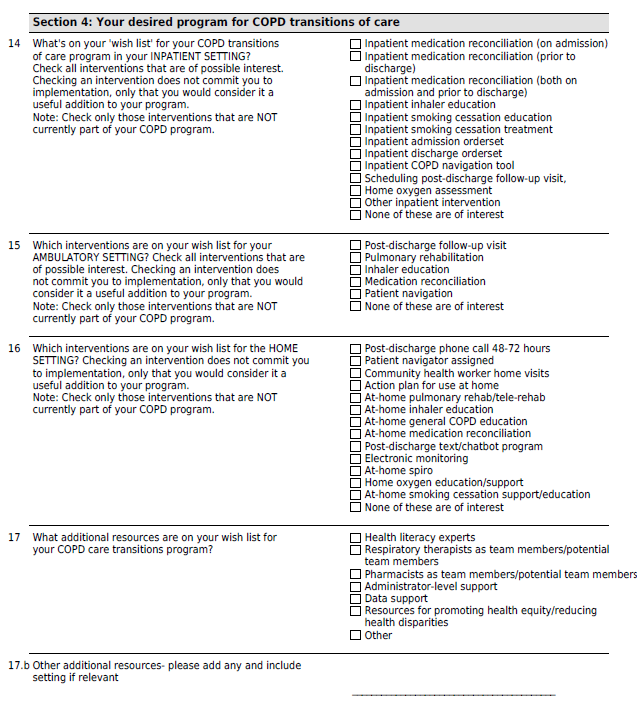
**
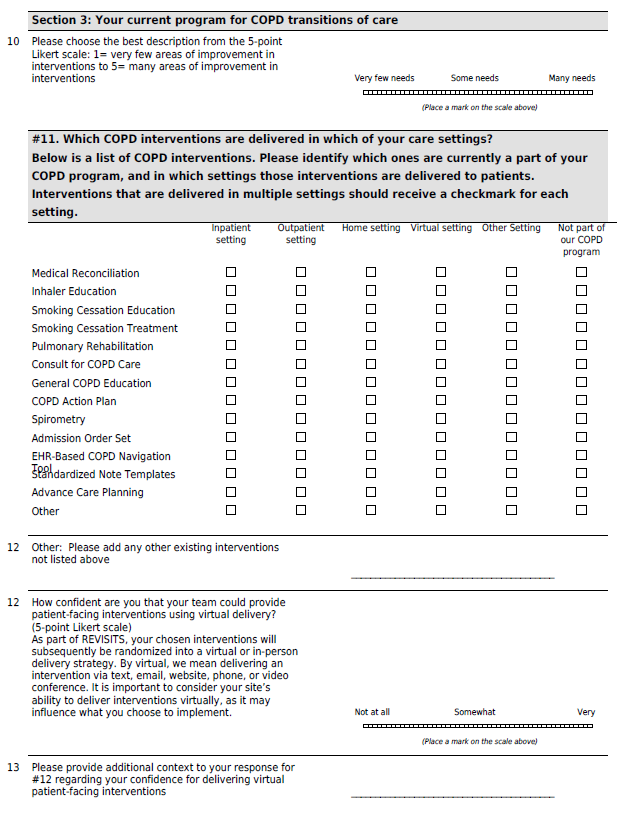

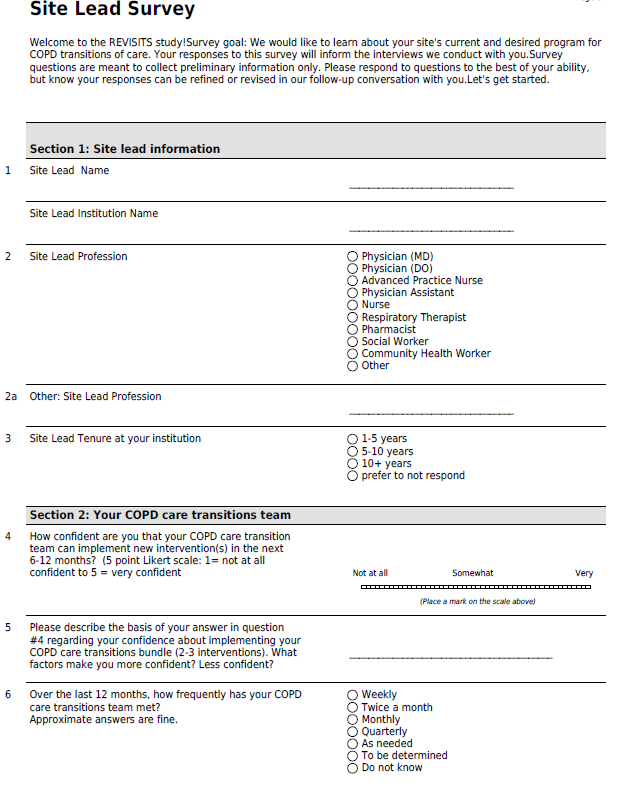

Supplement: Multimedia Appendix 1 [file humanfactors-v13-e82078-s001.docx]
