# Supplementary material for: Contextual Assessments for Chronic Obstructive Pulmonary Disease Transition of Care Bundle Implementation Planning for the Reduce REVISITS Study: Rapid Sequential Explanatory Mixed Methods Approach
Source: JMIR Hum Factors. 2026 Mar 2;13:e82078. doi: 10.2196/82078 (PMC12954717; doi:10.2196/82078)
Supplement: Multimedia Appendix 2 [file humanfactors-v13-e82078-s002.docx]

**Appendix B: Example Site Implementation Plan Workbook***

**
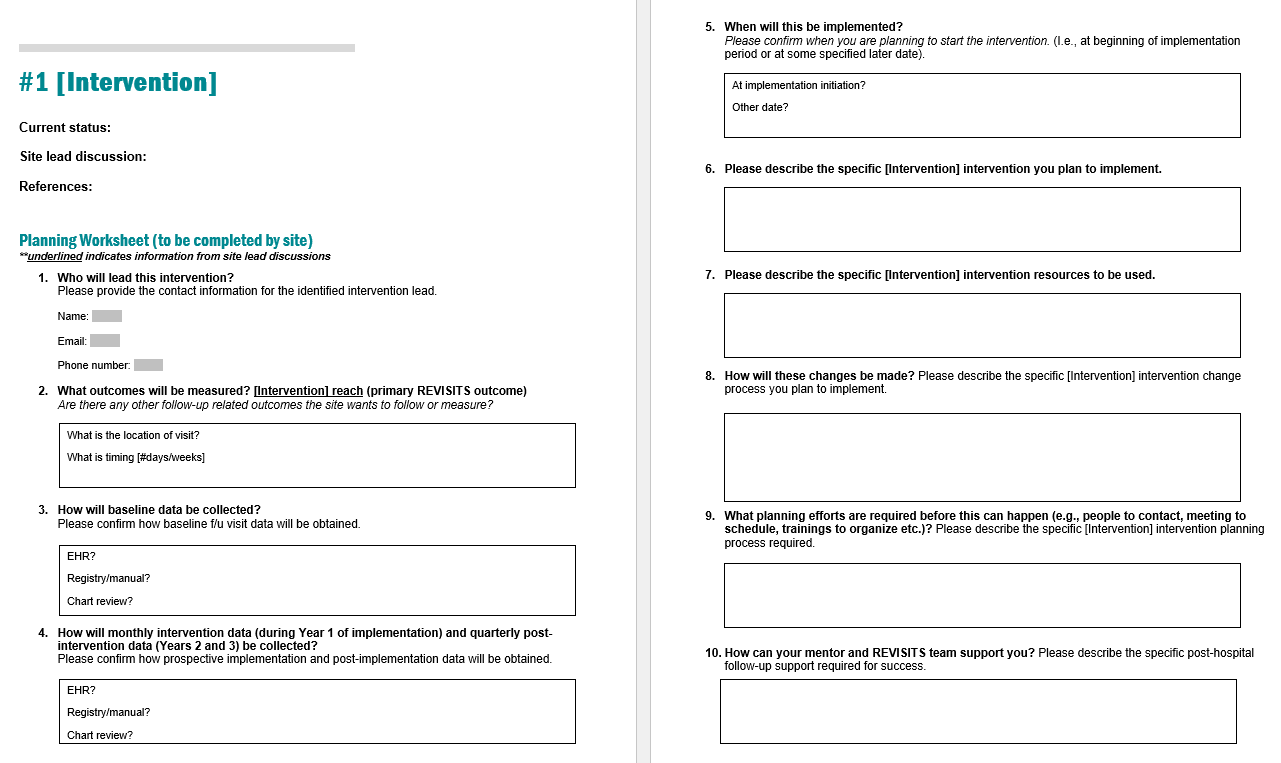
**

**The items represented in this example were repeated for all of the interventions included in the site’s COPD Transition of Care Bundle*
