# Supplementary material for: Contextual Assessments for Chronic Obstructive Pulmonary Disease Transition of Care Bundle Implementation Planning for the Reduce REVISITS Study: Rapid Sequential Explanatory Mixed Methods Approach
Source: JMIR Hum Factors. 2026 Mar 2;13:e82078. doi: 10.2196/82078 (PMC12954717; doi:10.2196/82078)
Supplement: Multimedia Appendix 3 [file humanfactors-v13-e82078-s003.docx]

**Supplementary Table S1.** Participant demographics of hospitalized adults with COPD across sites participating in the Reduce REVISITS study. Values represent aggregated demographic variables provided by sites with available data during the baseline period (N=10,697) and the implementation period (N=2,745). Demographic indicators include age, gender identity, race, ethnicity, insurance coverage, and smoking status. Only sites that submitted demographic data are included.

***Demographic DATA Across Sites****

****Those sites providing data***

|  | ***Study Period*** | |
| --- | --- | --- |
|  | ***Baseline (N_baseline_ = 10,697)*** | ***Implementation (Q1-Q2) (N_Implementation_ = 2,745)*** |
| **Age*** | |  |
| Mean (SD) | 67.03 (1.85) | 68.48 (5.03) |
| Median (Range) | 67.75 (18– 108.78) | 68 (18-99.70) |
| **Gender Identity*** | |  |
| Female | 5,757 (53.82%) | 1,455 (53.01%) |
| Male | 4,745 (44.35%) | 1,137 (41.42%) |
| Non-Binary | 0001 (0.0093%) | 0 (0.00%) |
| Missing | 000194 (1.81%) | 153 (5.57%) |
| **Race*** | |  |
| Asian | 169 (1.58%) | 42 (1.53%) |
| Black | 2,859 (26.73%) | 582 (21.20%) |
| Other/Unknown | 308 (2.88%) | 47 (1.71%) |
| White | 6,521 (60.96%) | 1,767 (64.37%) |
| Missing | 840(7.85%) | 307 (11.18%) |
| **Ethnicity*** | |  |
| Hispanic | 273(2.55%) | 147 (5.36%) |
| Not Hispanic | 10,048 (93.93%) | 2,142 (78.03%) |
| Other/Unknown | 107 (1.00%) | 48 (1.75%) |
| Missing | 269 (2.51%) | 408 (14.86%) |
| **Insurance*** | | |
| Dual Medicaid/Medicare | 98 (0.92%) | 106 (3.86%) |
| Medicaid | 1,565 (14.63%) | 464 (16.90%) |
| Medicare | 5,296 (49.51%) | 1,449 (52.79%) |
| Other/Unknown | 2,728 (25.50%) | 129 (4.70%) |
| Private | 640 (5.98%) | 329 (11.99%) |
| Uninsured | 172 (1.61%) | 35 (1.28%) |
| Missing | 198 (1.85%) | 233 (8.49%) |
| **Smoking*** | | |
| Current | 1,396 (13.05%) | 457 (16.65%) |
| Former | 2,075 (19.40%) | 605 (22.04%) |
| Never | 653 (6.10%) | 286 (10.42%) |
| Unknown/NULL | 3,348 (31.30%) | 509 (18.54%) |
| Missing | 3,225 (30.15%) | 888 (32.35%) |
